# Predictive Factors of Initial Pain and Disability in Patients Presenting With Neck Pain for Rehabilitation

**DOI:** 10.7759/cureus.87361

**Published:** 2025-07-05

**Authors:** Emmanouil D Kapernaros, Maria Moutzouri, Georgios Krekoukias, Nikolaos Chrysagis, George A Koumantakis

**Affiliations:** 1 Physiotherapy Department, School of Health and Care Sciences, University of West Attica (UNIWA), Athens, GRC; 2 Laboratory of Advanced Physiotherapy, Physiotherapy Department, School of Health and Care Sciences, University of West Attica (UNIWA), Athens, GRC

**Keywords:** anxiety, biopsychosocial model, coping mechanisms, depression, disability, neck pain, pain intensity, physical activity, physiotherapy, predictive factors

## Abstract

Background

Neck pain is a complex and multifactorial condition with increasing global prevalence and a substantial impact on disability. Understanding the interplay between physical and psychological factors at the onset of rehabilitation is critical for effective, individualized care.

Objective

To identify physical (body weight/height, pain chronicity, physical activity, posture) and psychological (anxiety, depression, job satisfaction) predictors of pain intensity and disability in patients with neck pain presenting for physiotherapy, aiming to enhance early-stage assessment and address a research gap regarding predictors at the point of initial physiotherapy evaluation.

Methods

A cross-sectional cohort study was conducted involving 71 patients with neck pain attending a physiotherapy clinic. Clinical assessments and validated questionnaires were used to evaluate physical and psychological parameters, including the Neck Disability Index (NDI), Numeric Rating Scale for pain (NRS), Hospital Anxiety and Depression Scale (HADS), International Physical Activity Questionnaire (IPAQ), and the Minnesota Satisfaction Questionnaire - Short Form (MSQ-Short). Stepwise multiple regression analyses were performed to identify predictors of pain intensity and disability.

Results

Pain intensity was positively associated with disability (r = 0.41, p < 0.001) and depression (r = 0.27, p < 0.001), but negatively associated with anxiety (r = −0.18, p = 0.012). Disability showed strong associations with pain intensity (r = 0.41, p < 0.001), depression (r = 0.54, p < 0.001), and anxiety (r = 0.34, p < 0.001). In the multivariable analysis, lower anxiety levels significantly predicted higher pain intensity (β = −0.134, p < 0.001), an unexpected finding that may reflect coping-related mechanisms. Higher disability was predicted by greater depression (β = 0.257, p < 0.001) and lower job satisfaction (β = −0.215, p < 0.001). The models explained 37.7% of the variance in pain intensity and 49.3% in disability.

Conclusion

Both physical and psychological factors significantly influence pain and disability in patients with neck pain at the initial physiotherapy evaluation. Early identification of these predictors may improve clinical decision-making and rehabilitation outcomes. Future longitudinal studies in broader populations are recommended to validate these findings.

## Introduction

Neck pain is a prevalent and disabling musculoskeletal condition, characterized by a complex interplay of structural, neurophysiological, and psychosocial mechanisms [1-4]. It ranks fourth globally in years lived with disability, with its burden projected to increase further due to population aging and occupational strain [1,5]. In Greece, the 12-month prevalence of neck pain reaches 29%, consistent with international trends [6].

This growing burden underscores the need to refine rehabilitation strategies and improve understanding of the physical and psychological contributors to neck pain-related disability. Prior research has identified several biopsychosocial predictors, including sex, age, heredity, physical activity (PA) level, sleep disorders, anxiety, depression, perceived health, coping ability, and kinesiophobia [7-10]. Emerging evidence suggests that lower job satisfaction may contribute to neck pain risk through occupational stress mechanisms, especially in working populations [10-12]. This reflects the broader biopsychosocial framework of musculoskeletal pain, which emphasizes the interactive effects of psychological and physical domains on pain perception and disability.

Recent studies emphasize the importance of assessing psychosocial factors in musculoskeletal pain populations, including neck pain, highlighting the significant associations between psychological and physical variables, and calling for integrated assessment approaches at the initial physiotherapy evaluation [13,14]. This study aimed to identify physical (body weight (BW), body height, pain chronicity, PA, posture) and psychological (anxiety, depression, job satisfaction) predictors associated with two key outcomes, pain intensity and disability, in patients presenting with neck pain at the initial physiotherapy evaluation. These outcomes were selected due to their central importance in capturing the patient experience and guiding treatment decisions in neck pain [3,15,16]. Pain intensity reflects the severity of symptoms and is a primary motivator for individuals to seek healthcare. In contrast, disability provides a more comprehensive measure of functional limitation and is essential for informing rehabilitation strategies and long-term care planning.

To our knowledge, there is no systematic review that explicitly investigates the interaction or association between psychological and physical predictors assessed at the initial physiotherapy evaluation in patients with neck pain. The present study addresses this gap, in combination with recent primary studies that have examined psychological and physical factors in neck pain populations [9,13], by analyzing a broader set of cross-sectional associations at the initial clinical presentation in a primary care context.

Addressing a previously underexplored area, this study contributes to the development of more precise assessment and early intervention strategies in musculoskeletal rehabilitation.

## Materials and methods

Participants

The study sample comprised patients who sought physiotherapy for neck pain and associated disability at a private physiotherapy clinic in Attica, Greece. Participants were either referred by a physician or self-referred. All participants provided written informed consent after receiving a comprehensive explanation of the study’s purpose, methodology, and procedures. Participation was entirely voluntary. Inclusion and exclusion criteria were adapted from a previous study investigating physiotherapy outcomes in patients with neck pain [17].

Inclusion criteria were: (a) age between 18 and 65 years, and (b) current health status consistent with a diagnosis of nonspecific neck pain, at any stage (acute, subacute, or chronic), and/or unilateral or bilateral cervical radiculopathy.

Exclusion criteria included: medical history suggesting serious cervical pathology ("red flags"), symptoms indicating a non-musculoskeletal or non-psychological etiology, or any condition requiring urgent medical referral. Additional exclusion criteria were: history of cervical surgery or trauma, osteoporosis, diagnosed mental health disorders, tumor history, pregnancy, cerebrovascular or neurological conditions, autoimmune diseases related to neck pain, and inability to read or understand Greek. Patients exhibiting signs of vertebrobasilar artery insufficiency or upper cervical ligamentous instability were also excluded [18]. No formal diagnostic criteria were applied for the inclusion of patients with nociplastic pain, as such definitions are still under development [14,19].

The study received ethical approval from the Ethics Committee of the University of West Attica (Approval No. 17778/11-03-2024) and was conducted in accordance with the principles of the Declaration of Helsinki.

Study design

This was a cross-sectional observational study, modeled on prior studies employing similar methodology [13,20]. Data collection took place between March and August 2024 at the physiotherapy clinic where the study was conducted. All data were securely stored in anonymized document records accessible only to the research team, away from the clinic. The study involved clinical examination and administration of self-reported questionnaires, conducted in a private, controlled setting. The clinical examination included physical assessment and photogrammetry, while the self-reported questionnaires included demographic information and the Greek versions of the Neck Disability Index (NDI), the Numeric Rating Scale for pain intensity (NRS - pain intensity), the International Physical Activity Questionnaire - Short Form (IPAQ-SF), the Hospital Anxiety and Depression Scale (HADS), and the Minnesota Satisfaction Questionnaire - Short Form (MSQ-Short).

All clinical assessments were performed by licensed physiotherapists employed at the clinic, each with a minimum of five years of clinical experience, including the principal investigator, who has over 18 years of experience in musculoskeletal rehabilitation. Self-reported questionnaires were completed by participants on-site under supervision.

Outcome measures

The outcome measures in this study were pain intensity and disability level. These outcomes were selected based on prior cohort studies involving patients with neck pain [7,13,16,17,20-23].

All psychometric tools used in this study were validated Greek versions. Permission for the use of the Hospital Anxiety and Depression Scale (HADS) was obtained under formal request from the Mapi Research Trust, which manages licensing for the original instrument developed by Zigmond and Snaith (1983). The validated Greek version by Michopoulos et al. (2008) was used in its original form without modifications [24]. The instrument was applied solely for academic, non-commercial research purposes in accordance with the licensing terms. For all other instruments, use was unrestricted for academic purposes upon proper citation of the respective sources and Greek adaptations.

Disability: Neck Disability Index

The NDI is a widely used self-report questionnaire for evaluating neck-related functional disability [5,21,25-29]. The Greek version of the NDI was used in this study, which has been previously validated with excellent test-retest reliability (ICC = 0.93) and good internal consistency (Cronbach’s α = 0.85) [30,31].

The instrument comprises 10 items rated on a 6-point Likert scale. It assesses the following domains: pain intensity, personal care, lifting, reading, headaches, concentration, work, driving, sleeping, and recreation. Total scores range from 0 to 50 and are interpreted as follows: no disability (0-4), mild (5-14), moderate (15-24), severe (25-34), and complete disability (35-50) [25,30].

Pain Intensity: Numeric Rating Scale

The NRS-pain intensity was used to measure average neck pain intensity over the previous week. This 11-point scale ranges from 0 (“no pain”) to 10 (“worst imaginable pain”) and has demonstrated superior patient compliance compared to visual analog scales (VAS) [32].

It is a valid, reliable, and responsive tool for assessing neck pain [33,34]. It is easy to administer and analyze, with a clinically significant change ranging from 1.5 to 4.1 points, depending on the intervention, evaluation purpose, and clinical context.

Independent variables: predictive factors

Physical Predictive Factors

Demographics: Demographic information included age, sex, BW, and body mass index (BMI). Participants were also asked to report the chronicity of their symptoms (in months), the location of symptoms (confined to the neck and/or radiating to the upper limbs), and their current pain intensity using the 11-point NRS (NRS - pain intensity).

Clinical assessment and medical history: Clinical assessment was conducted according to the International Federation of Orthopaedic Manipulative Physical Therapists (IFOMPT) guidelines [35], and informed by previous studies involving manual therapy in the cervical spine, in order to confirm that the patient can undergo manual therapy interventions, thus included in the study [17,36].

International Physical Activity Questionnaire (short form): The Greek version of the IPAQ-SF was used to assess participants’ PA levels, providing an objective estimate of daily PA [22,37,38]. It was used as a categorical variable (Low, Moderate, High PA) in the regression analysis, as per the scoring protocol.

Metabolic equivalent (MET) scores were calculated to classify PA level. Only responses with valid time and frequency entries (10-180 minutes per day) were retained. Responses such as “I don’t know” or missing duration were excluded [39].

The Greek IPAQ-SF has demonstrated high test-retest reliability over a nine-day interval, particularly for total and vigorous PA (ICC = 0.84-0.93), and moderate reliability for sedentary time (ICC = 0.69), vigorous PA (ICC = 0.81), and walking (ICC = 0.75). Inter-rater reliability was acceptable to high (ICC = 0.70-0.87), though lower for moderate PA (ICC = 0.58-0.64). Validity is supported by significant correlations with maximum treadmill walking time (r = 0.35-0.43) [39]. 

Forward head posture in seated working position - photogrammetry: Forward head posture was assessed via photogrammetric measurement of the craniovertebral angle (CVA) using the PostureScreen Mobile® application (PostureCo Inc., Trinity, Florida). This validated tool demonstrates high inter-rater (r = 0.88) and test-retest reliability (r = 0.92) in healthy adults, based on measurements performed in a standing posture among asymptomatic individuals. [40]. Intra-rater reliability has also been reported for angular measurements related to CVA, such as head tilt and shoulder tilt, with ICC values of 0.78 using repeated assessments [41]. Additional data from comparisons with three-dimensional motion analysis systems show that head tilt and shift measurements obtained with PostureScreen Mobile® achieve ICC values up to 0.66 and angular bias within 5°, which is considered clinically acceptable [42].

Assessment took place in a simulated workstation environment. Participants were asked to sit at a desk and adjust the chair, monitor, keyboard, and mouse to their personal comfort, reflecting the posture they would typically adopt if they were to work at that station. The measurement was conducted in this self-selected, habitual computer-use position [43]. 

**Figure 1:**
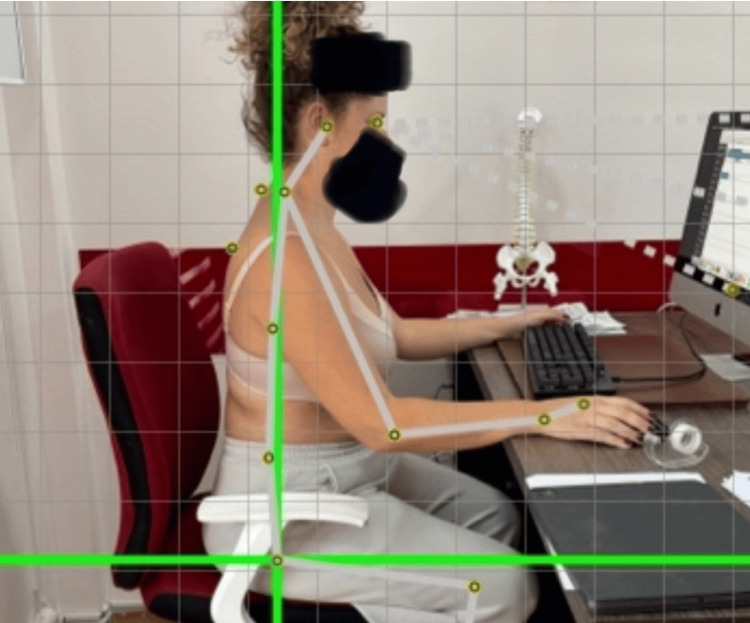
Photogrammetric assessment of the craniovertebral angle (CVA) using the PostureScreen Mobile® (PostureCo Inc., Trinity, Florida) application in a simulated seated workstation setup. Reflective markers are placed on anatomical landmarks (the tragus and the spinous process of C7) and image capture is performed with the participant in a self-selected, habitual working posture.

A smartphone (iPhone, Apple Inc.) was placed 3 meters away at shoulder height to capture lateral images. Participants wore minimal upper body clothing to ensure clear visualization of anatomical landmarks [41]. Reflective markers were applied to the tragus and spinous process of C7, and the CVA was calculated from the lateral image. The procedure is illustrated in Figure 1, which demonstrates the participant’s seated posture, marker placement on anatomical landmarks, and the grid-based photogrammetric analysis used for CVA estimation. A CVA greater than 53° was considered within normal limits, while values below 45° indicated severe postural deviation [44].

Detailed anatomical landmark identification and standardized protocol for posture capture and measurement were employed according to the Sit Screen module of the PostureScreen Mobile® application, ensuring reproducibility and reliability.

Psychological Predictive Factors

Hospital Anxiety and Depression Scale (HADS): HADS is a widely used self-report instrument designed to assess emotional distress, anxiety, and depression in individuals with physical illness [45,46]. It has been applied in primary healthcare settings for patients with neck pain [47], as well as in studies evaluating interventions targeting psychological symptoms related to neck disorders [48,49].

HADS consists of 14 items, with 7 items each for anxiety (HADS-A) and depression (HADS-D), rated on a scale of 0 to 21 per subscale. The Greek version has been validated in clinical and community samples (n = 521), showing excellent internal consistency (Cronbach’s α = 0.870-0.885) and very high test-retest reliability (r = 0.994) [24]. Structural validity is supported by item loadings (0.540-0.804) and significant correlations with the State-Trait Anxiety Inventory (STAI; r = 0.628, p < 0.001) and the Beck Depression Inventory (BDI; r = 0.661, p < 0.001).

Score interpretation per subscale is as follows: normal (0-7), mild (8-10), moderate (11-14), and severe psychological distress (15-21) [45].

Minnesota Satisfaction Questionnaire - Short Form (MSQ-Short): MSQ-Short is a 20-item self-report instrument used to assess both intrinsic (task-related) and extrinsic (context-related) job satisfaction [50]. Each item is rated on a 5-point Likert scale ranging from 1 (“very dissatisfied”) to 5 (“very satisfied”).

The Greek version has demonstrated strong psychometric properties and is considered valid, brief, and easy to administer in healthcare contexts [51,52]. Total scores range from 20 to 100, with higher scores indicating greater levels of job satisfaction [52].

Procedures

All patients presenting to the private physiotherapy clinic with complaints of neck pain were initially informed about the study by the clinic’s front-desk personnel. Those who expressed interest in participating were provided with a written informed consent form, accompanied by a coded sheet containing their reference number (corresponding to the clinic’s internal patient database), and sections to record demographic information, the NRS-pain score, and the CVA data. Paper versions of all study questionnaires were also included in the initial materials.

Subsequently, the patient was escorted to a private consultation room to begin the initial physiotherapy evaluation, upon signing the consent form. A licensed physiotherapist available at the time conducted the assessment. The clinical evaluation included measurement of active cervical range of motion, palpation for segmental stiffness, pain reproduction testing via passive intervertebral motion, and screening for red flags, including vertebrobasilar insufficiency and upper cervical instability. These procedures ensured clinical homogeneity at baseline.

Following the clinical assessment, participants were asked to complete their demographic information, including sex, age, BW, height, duration of symptoms (in months), occupational status (office-based or not), and presence or absence of referred pain to the upper limb. They then completed the NRS pain scale. Next, photogrammetric analysis of the CVA was performed in a standardized seated workstation setup, as previously described.

Finally, participants were instructed to complete the study questionnaires: NDI, HADS, IPAQ-SF, and the MSQ-Short. A physiotherapist remained available throughout the process to provide clarification or assistance if needed.

Upon completion, all documents were returned to the clinic reception. Each participant’s materials were placed into a sealed envelope labeled only with their unique reference number. These envelopes were stored in a locked cabinet at the clinic until collected by the principal investigator. All study materials were then transferred to a secure, locked storage unit accessible exclusively to the research team to ensure confidentiality and compliance with data protection regulations.

Statistical analysis

All statistical analyses were conducted using IBM SPSS Statistics for Windows, Version 29 (Released 2023; IBM Corp., Armonk, New York).

Normality Testing

Normality of distribution for all continuous variables was assessed using the Shapiro-Wilk test, which is preferred over the Kolmogorov-Smirnov test for small-to-moderate sample sizes [53]. 

Due to missing responses in 15 participants (21.1% of the sample), normality testing for the IPAQ data was conducted separately, in accordance with the questionnaire’s guidelines for partial completion [39]. This approach ensured that the normality assessment of the remaining variables was not compromised by incomplete IPAQ data. 

There were no missing values in the remaining variables; therefore, complete case analysis was applied.

Demographic Data

Demographic characteristics, questionnaire-based data, and subjective clinical assessments were summarized using descriptive statistics. Categorical variables were expressed as absolute frequencies and percentages. Continuous variables were presented as means ± standard deviations (SD) for normally distributed data, or as medians with interquartile ranges (IQR) for non-normally distributed data.

Identification of Predictive Factors for Initial Levels of Pain and Disability - Univariate Analysis

To establish a clinical profile of the study population, associations between pain intensity and disability and a range of physical and psychological variables were examined using univariate analyses, consistent with prior research [7,8,21-23,54,55]. A p-value of ≤ 0.05 was considered statistically significant.

Univariate analyses were performed to explore basic relationships between the variables without adjusting for potential confounders [56]. To account for such confounding effects and to identify a parsimonious set of predictive factors for pain and disability, multivariable regression analysis was subsequently conducted [56,57]. No correction for multiple comparisons was applied during univariate testing due to the exploratory nature of the study and the limited sample size, which could increase the chance of type I errors.

The minimum required sample size for multivariable modeling was determined using the empirical ratio of 10 participants per predictor variable. This rule-of-thumb approach has been widely used in clinical prediction research [17,20,58,59].

Categorical variables: To explore associations between categorical variables and the outcomes of pain intensity and disability, non-parametric statistical tests were employed. For variables with two independent groups, the Mann-Whitney U test was used. For variables with more than two categories, the Kruskal-Wallis test was applied.

These tests were selected due to the non-normal distribution of outcome variables and the ordinal or non-normally distributed nature of the independent categorical predictors.

Quantitative variables: Correlation analysis was performed to examine associations between quantitative independent variables and the dependent outcomes of pain intensity and disability. Given the presence of both normally and non-normally distributed variables, Spearman’s rank-order correlation coefficient (Spearman’s ρ) was used, as it is appropriate for assessing monotonic relationships involving non-parametric data [60].

Multivariable Analysis

Stepwise multiple linear regression analysis was conducted to identify significant predictors of pain intensity and disability at initial physiotherapy evaluation. This method sequentially adds and removes independent variables based on statistical significance, retaining only those that meaningfully contribute to the final model [61]. 

All assumptions for linear regression were tested and met for both models, including normality of residuals (via histogram and P-P plot), homoscedasticity (via scatterplot of standardized residuals), and absence of multicollinearity (confirmed by VIF < 2). Standardized beta coefficients were calculated to facilitate the interpretation of predictor importance.

Model performance was assessed using the adjusted R², which accounts for the number of predictors and helps mitigate overfitting by adjusting for model complexity [59]. All clinically and theoretically relevant quantitative variables were entered into the model, irrespective of their statistical significance in univariate analyses, following best practices in multivariable modeling [62,63]. 

The commonly accepted “1:10 rule”, which requires at least 10 participants per predictor, was applied to the final model rather than the initial pool of variables. Although resampling methods (e.g., bootstrap or cross-validation) were not used, simulation studies suggest that stepwise regression can yield reliable estimates and adjusted R² values, even with fewer than 10 subjects per variable, when overfitting is appropriately managed [59,63].

Stepwise regression enhances model parsimony by excluding non-contributory predictors while retaining those with significant explanatory power [64]. Theoretical justification for variable inclusion remains essential in predictive modeling, particularly in smaller datasets where overfitting risk is elevated [62]. 

In this study, the final models for pain and disability each included only two predictors, maintaining a subject-to-variable ratio considered acceptable even under conservative modeling standards.

## Results

**Figure 2:**
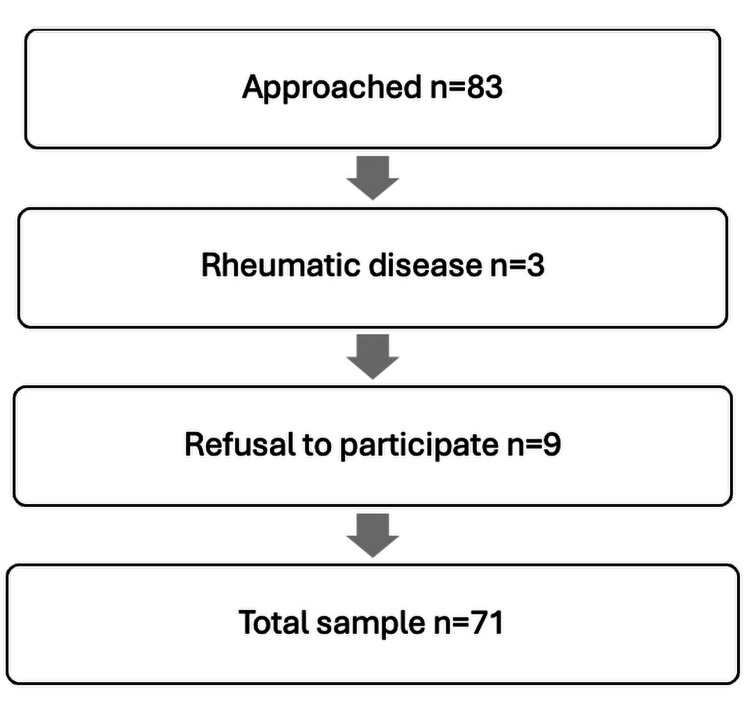
Patient flow diagram

Between March and August 2024, a total of 83 patients were approached for participation in this study. Of these, 12 were excluded: 3 due to the presence of rheumatic disease and 9 who declined participation. Consequently, 71 patients (52 women) were included in the final data analysis. Figure 2 illustrates the patient inclusion flow.

Normality test of variables

The Shapiro-Wilk (S-W) test revealed that not all variables followed a normal distribution. Specifically, BW, HADS (total and subscales), MSQ, and CVA were normally distributed. In contrast, the remaining variables exhibited significant deviations from normality, warranting the use of non-parametric statistical tests in subsequent analyses.

Identification of predictive factors for pain and disability

Demographic Data

The mean age of participants was 42.66 years (IQR: 34-51). Age group distribution was as follows: 10 participants (14.1%) were aged 20-30 years, 21 (29.6%) aged 30-40 years, 19 (26.8%) aged 40-50 years, 11 (15.5%) aged 50-60 years, and 10 (14.1%) were over 60 years old. The sample consisted of 19 men (26.8%) and 52 women (73.2%).

The mean height was 1.68 m (IQR: 1.62-1.75 m), and the mean BW was 75.38 kg (SD: ±18.35). The average BMI was 26.26 kg/m² (IQR: 22.14-28.40). Based on BMI classification, 3 participants (4.2%) were underweight, 27 (38.0%) had normal weight, 28 (39.4%) were overweight, and 13 (18.3%) were obese. 

Medical History Related to Neck Pain

Based on participants’ medical histories, 30 individuals (42.3%) were classified as being in the acute or subacute stage of neck pain, having experienced symptoms for up to two months. The remaining 41 participants (57.7%) presented with chronic symptoms, defined as persisting for three months or longer.

In terms of symptom location, 44 participants (62.0%) reported localized neck pain without radiation to the upper limbs, whereas 27 (38.0%) reported referred pain radiating to one or both upper limbs.

Pain and Disability 

The mean pain intensity score was 7.11 on a 0-10 NRS-pain intensity, with an IQR of 6-8. The mean NDI score was 13.03 (scale range: 0-50), with an IQR of 8-17.

Based on NDI classification, 3 participants (4.2%) were categorized as having no disability, 44 (62.0%) as mild, 17 (23.9%) as moderate, 6 (8.5%) as severe, and 1 participant (1.4%) as having complete disability.

Physical Factors

The mean weekly MET expenditure was 2,248.23 MET-minutes (IQR: 675-2,860.25). Participants reported an average sedentary time of 7.87 hours per day (IQR: 4-10 hours). Based on PA classification, 26 participants (36.6%) had low PA levels, 28 (39.4%) had moderate PA levels, and 17 (23.9%) were classified as highly active.

The CVA had a mean value of 42.29° (SD: ±9.99). According to predefined postural thresholds, 6 participants (8.5%) had a normal CVA, 28 (39.4%) had postural deviation, and 37 (52.1%) demonstrated severe deviation.

Occupational data revealed that 42 participants (59.2%) worked in an office-based setting, while 29 (40.8%) were employed in non-office-based occupations.

Psychological Factors

The mean anxiety score, as measured by the HADS - Anxiety subscale (HADS-A), was 7.87 (SD: ±3.71) on a scale from 0 (no anxiety) to 21 (maximum anxiety). Based on standard cutoff scores, 34 participants (47.9%) were classified as normal, 25 (35.2%) as mildly anxious, 10 (14.1%) as moderately anxious, and 2 (2.8%) as severely anxious.

The mean depression score, HADS - Depression (HADS-D), was 6.07 (SD: ±3.34), also on a 0-21 scale. According to HADS-D thresholds, 49 participants (69.0%) were categorized as normal, 14 (19.7%) had mild depression, 7 (9.9%) had moderate depression, and 1 (1.4%) had severe depression.

Job satisfaction, assessed using the MSQ-Short, had a mean score of 44.93 (SD: ±15.00), on a scale where 20 indicates complete satisfaction and 100 indicates maximum dissatisfaction. In terms of employment status, 63 participants (88.7%) reported being employed by an organization, while 8 (11.3%) were self-employed.

An overview of all baseline characteristics across demographic, physical, and psychological domains is presented in Table 1.

**Table 1:** Descriptive characteristics of patients with neck pain (n=71) BW: body weight, BMI: body mass index, NDI: Neck Disability Index, NRS: Numeric Rating Scale, HADS: Hospital Anxiety and Depression Scale, HADS-A: HADS - Anxiety Subscale, HADS-D: HADS - Depression Subscale, IPAQ: International Physical Activity Questionnaire, MSQ: Minnesota Satisfaction Questionnaire, METs: metabolic equivalents, CVA: craniovertebral angle.

Characteristics	n (%)	SD or IQR
Sex		
Male	19 (26.8%)	
Female	52 (73.2%)	
Age (years)		42.66 (34–51)
20–30	10 (14.1%)	
31–40	21 (29.6%)	
41–50	19 (26.8%)	
51–60	11 (15.5%)	
>60	10 (14.1%)	
Height (meters)		1.68 (1.62–1.75)
Body weight (kg)		75.38 (18.35)
BMI		26.26 (22.14–28.4)
Underweight	3 (4.2%)	
Normal	27 (38%)	
Overweight	28 (39.4%)	
Obese	13 (18.3%)	
Chronicity (months)		4 (2–7)
Acute – Subacute	30 (42.3%)	
Chronic	41 (57.7%)	
Office work		
Yes	42 (59.2%)	
No	29 (40.8%)	
Location of the symptoms		
No referred pain	44 (62%)	
Referred pain	27 (38%)	
NRS – pain intensity		7.11 (6–8)
NDI		13.03 (8–17)
No disability	3 (4.2%)	
Mild disability	44 (62%)	
Moderate disability	17 (23.9%)	
Severe disability	6 (8.5%)	
Total disability	1(1.4%)	
IPAQ category		
Low	26 (36.6%)	
Moderate	28 (39.4%)	
High	17 (23.9%)	
IPAQ METs/week		2248.23 (675–2860.25)
IPAQ sitting h/day		7.29 (4–10)
HADS-A		7.87 (3.71)
Normal	34 (47.9%)	
Mild	25 (35.2%)	
Moderate	10 (14.1%)	
Severe	2 (2.8%)	
HADS-D		6.07 (3.34)
Normal	49 (69%)	
Mild	14 (19.7%)	
Moderate	7 (9.9%)	
Severe	1 (1.4%)	
HADS total		13.94 (6.12)
MSQ		44.93 (15)
Employment status		
Employee	63 (88.7%)	
Self-employed	8 (11.3%)	
CVA		42.29 (9.99)
Normal	6 (8.5%)	
Deviation	28 (39.4%)	
Severe deviation	37 (52.1%)	

Physical and Psychological Factors Affecting Pain and Disability

Pain: Analysis of NRS-pain intensity in relation to categorical physical and psychological variables revealed statistically significant differences across several factors. Patients reporting referred pain to the upper limbs experienced higher pain levels compared to those without referred pain (p = 0.029). Similarly, significant differences in pain intensity were observed based on disability severity (NDI categories; p < 0.001), with individuals in the severe (NDI 25-34/50) and complete disability groups (NDI >34/50) reporting the highest levels of pain.

PA level (IPAQ categories) was also significantly associated with pain intensity (p = 0.011). Interestingly, patients classified as having high PA reported the highest pain levels, while those with moderate PA reported the lowest (Table 2).

**Table 2:** Associations of NRS – pain intensity with the categorical variables of the sample characteristics. NDI: neck disability index, BMI: body mass index, IPAQ: International Physical Activity Questionnaire, HADS-A: Hospital Anxiety and Depression Scale – Anxiety Subscale, HADS-D: Hospital Anxiety and Depression Scale – Depression Subscale, MSQ: Minnesota Satisfaction Questionnaire – Short Form, CVA: craniovertebral angle.

Characteristics	n	Mean NRS – pain intensity	IQR	Minimum–maximum	Mann-Whitney U or Kruskal-Wallis p (Bonferroni)
Sex					0.112
Male	19	6.74	6-8	3-9	
Female	52	7.25	6-9	1-10	
Age (years)					0.961
20–30	10	7.1	5.75-9	3-10	
31–40	21	7.24	7-8.5	1-10	
41–50	19	6.95	5-9	3-10	
51–60	11	7.27	6-9	3-10	
>60	10	7	6-8	4-9	
BMI					0.645
Underweight	3	6	-	3-9	
Normal	27	7.41	6-9	4-10	
Overweight	28	7.14	6.25-8	1-10	
Obese	13	6.69	5-8.5	3-9	
Chronicity (months)					0.251
Acute–subacute	30	7.37	7-8.25	1-10	
Chronic	41	6.93	6-8.5	3-10	
Office work					0.686
Yes	42	7.1	6-8	3-10	
No	29	7.14	6-9	1-10	
Location of the symptoms					0.029
No referred pain	44	6.8	6-8	3-9	
Referred pain	27	7.63	7-9	1-10	
NDI					<0.001 (a-c,a-d,b-d)
No disability (a)	3	3.67	-	3-4	
Mild disability (b)	44	6.7	6-8	1-10	
Moderate disability (c)	17	8	7-9	5-10	
Severe disability (d)	6	9	8-10	8-10	
Total disability (e)	1	9	-	-	
IPAQ category					0,011 (b-a,b-c)
Low (a)	26	7.5	7-9	1-10	
Moderate (b)	28	6.32	5-8	3-9	
High (c)	17	7.82	7-9	4-10	
HADS-A					0.716
Normal	34	7.26	6-9	3-10	
Mild	25	7	6-8	1-10	
Moderate	10	6.7	4.75-8.25	4-10	
Severe	2	8	-	7-9	
HADS-D					0.211
Normal	49	6.92	6-8	1-10	
Mild	14	7.14	6.5-8.25	3-9	
Moderate	7	8.43	7-9	7-10	
Severe	1	7	-	7	
MSQ					0.427
Employment status					
Employee	63	7.05	6-8	1-10	
Self-employed	8	7.63	6.25-9	5-9	
CVA (classification)					0.080
Normal	6	5.67	3.75-7.25	3-8	
Deviation	28	6.96	6-8	3-9	
Severe deviation	37	7.46	7-9	1-10	

Regarding continuous variables, pain intensity showed a moderate positive correlation with disability (r = 0.594, p < 0.001) and a weak but statistically significant positive correlation with depression scores (r = 0.240, p = 0.043) (Table 4).

Disability: Analysis of the NDI scores in relation to categorical physical and psychological variables revealed no statistically significant associations. None of the examined characteristics, including sex, age, symptom location, PA level, postural alignment (CVA), or psychological status, were significantly related to disability levels (Table 3).

**Table 3:** Associations of NDI with the categorical variables of the sample characteristics. NDI: neck disability index, BMI: body mass index, IPAQ: International Physical Activity Questionnaire, HADS-A: Hospital Anxiety and Depression Scale – Anxiety, HADS-D: Hospital Anxiety and Depression Scale – Depression, MSQ: Minnesota Satisfaction Questionnaire, CVA: craniovertebral angle.

Characteristics	n	Mean NDI	IQR	Minimum–maximum	Mann-Whitney U or Kruskal-Wallis p (Bonferroni)
Sex					0.289
Male	19	12.53	6-16	3-45	
Female	52	13.21	9-17	1-30	
Age (years)					0.961
20–30	10	13.3	9-18.5	6-21	
31–40	21	13.95	8.5-15	5-45	
41–50	19	12.68	6-17	2-30	
51–60	11	10.45	9-14	1-17	
>60	10	14.3	7.25-22.25	3-27	
BMI					0.402
Underweight	3	11.67	-	9-17	
Normal	27	13.3	9-17	5-27	
Overweight	28	14.14	8.25-18.5	2-45	
Obese	13	10.38	5.5-14	1-26	
Chronicity (months)					0.101
Acute–subacute	30	14.97	9-19.25	6-45	
Chronic	41	11.61	7-14.5	1-27	
Office work					0.251
Yes	42	11.98	8-15	2-30	
No	29	14.55	9-19	1-45	
Location of the symptoms					0.302
No referred pain	44	11.91	8-15.75	1-27	
Referred pain	27	14.85	9-21	6-45	
IPAQ category					0.377
Low (a)	26	13.23	9-13.25	5-45	
Moderate (b)	28	11.79	7.25-15.75	1-27	
High (c)	17	14.76	8.5-20.5	2-30	
HADS-A					0.213
Normal	34	11.59	7.75-15	1-30	
Mild	25	13.44	9-17	2-45	
Moderate	10	15.7	9-21	5-27	
Severe	2	19	-	12-26	
HADS-D					0.139
Normal	49	11.65	8-15	1-30	
Mild	14	14.5	9.75-19.25	5-27	
Moderate	7	19.86	11-27	6-45	
Severe	1	12	-	-	
MSQ					0.094
Employment status					
Employee	63	12.46	8-17	1-30	
Self-employed	8	17.5	11.25-16.75	11-45	
CVA (classification)					0.264
Normal	6	9.17	7.25-11.5	5-13	
Deviation	28	11.86	9-14.75	2-22	
Severe deviation	37	14.54	8-20	1-45	

Regarding quantitative variables, disability (NDI) demonstrated a moderate positive correlation with pain intensity (r = 0.594, p < 0.001), a moderate positive correlation with depression (HADS-D) scores (r = 0.435, p < 0.001), and a moderate correlation with the combined anxiety and depression score (HADS total; r = 0.387, p < 0.001). These associations are summarized in Table 4.

**Table 4:** Associations between the study’s quantitative variables using Spearman’s rho (n = 71). P-values are reported in parentheses. NDI: neck disability index, HADS: Hospital Anxiety and Depression Scale, HADS-A: Hospital Anxiety and Depression Scale – Anxiety, HADS-D: Hospital Anxiety and Depression Scale – Depression, IPAQ: International Physical Activity Questionnaire, MSQ: Minnesota Satisfaction Questionnaire, METs/week: metabolic equivalents per week, CVA: craniocervical angle, h/day: hours per day.

	Age	Body weight	Body height	Chronicity (months)	NRS -pain intensity	NDI	HADS-A	HADS-D	HADS	iPAQ METs/week	iPAQ sittingh/day	MSQ
Body weight	.147 (.221)	-	-	-	-	-	-	-	-	-	-	-
Body height	-.135 (.261)	.560 (.001)	-	-	-	-	-	-	-	-	-	-
Chronicity (months)	.237 (.047)	.045 (.712)	-.086 (.475)	-	-	-	-	-	-	-	-	-
NRS – pain intensity	.011 (.926)	-.158 (.189)	-.074 (.540)	-.119 (.324)	-	-	-	-	-	-	-	-
NDI	-.016 (.895)	-.118 (.326)	-.024 (.842)	-.139 (.247)	.594 (.001)	-	-	-	-	-	-	-
HADS-A	-.010 (.932)	.040 (.743)	.015 (.903)	.142 (.237)	-.058 (.629)	.224 (.061)	-	-	-	-	-	-
HADS-D	.035 (.771)	.163 (.175)	.069 (.565)	-.169 (.159)	.240 (.043)	.435 (.001)	.489(.001)	-	-	-	-	-
HADS	.004 (.974)	.114 (.345)	.056 (.642)	-.022 (.856)	.113 (.346)	.387 (.001)	.865(.001)	.849 (.001)	-	-	-	-
iPAQ METs/week	-.063 (.631)	.104 (.431)	.166 (.206)	-.177 (.175)	.190 (.146)	.205 (.115)	-.058(.658)	.042 (.749)	-.015 (.911)	-	-	-
iPAQ sitting h/day	-.187 (.136)	-.131 (.297)	-.051 (.687)	-.188 (.133)	-.054 (.670)	-.048 (.706)	.009(.943)	.006 (.965)	-.007 (.956)	.004 (.975)	-	-
MSQ	-.168 (.161)	-.018 (.880)	.022 (.858)	.055 (.651)	.111 (.358)	.006 (.962)	.203 (.090)	.237 (.046)	.253 (.034)	-.235 (.071)	.074 (.556)	-
CVA	-.041 (.736)	-.438 (.001)	-.003 (.982)	-.028 (.819)	-.110 (.361)	-.011 (.926)	.110 (.362)	-.055 (.647)	.020 (.868)	.018 (.891)	.156 (.214)	-.062 (.608)

Associations of Pain Intensity and Disability With Physical and Psychological Factors

Pain: A stepwise multiple linear regression analysis was conducted to identify significant predictors of pain intensity. The final model revealed that disability (NDI) was a strong positive predictor (β = 0.190, t = 5.929, p < 0.001), indicating that each one-point increase in NDI score was associated with a 0.19-point increase in pain intensity.

Α novel and counterintuitive finding emerged regarding psychological predictors. Contrary to prevailing literature linking anxiety to heightened pain sensitivity, our data revealed an inverse relationship between anxiety and reported pain intensity. HADS-A demonstrated a statistically significant negative association with pain intensity (β = −0.134, t = −2.185, p < 0.001), suggesting that higher anxiety scores were associated with slightly lower reported pain levels. Given the theoretical and clinical relevance of this deviation, this finding warrants further examination.

The intercept of the model (β = 5.479) represents the estimated baseline pain score when both NDI and anxiety are equal to zero, indicating that some level of pain may exist independently of disability or anxiety levels, possibly due to unmeasured factors.

The model explained 37.7% of the variance in pain intensity (adjusted R² = 0.377), indicating a moderate to strong level of explanatory power. All regression assumptions were satisfied for this model, including normal distribution of residuals, linearity, homoscedasticity, and absence of multicollinearity. Standardized beta coefficients are reported to facilitate the interpretation of predictor contribution (Table 5).

**Table 5:** Regression results for the variable NRS - pain intensity. NRS: Numeric Rating Scale, NDI: Neck Disability Index, HADS-A: Hospital Anxiety and Depression Scale – Anxiety subscale, SE: standard error, CI: confidence interval.

Variables	NRS - pain intensity				
	Unstandardized β (SE)	Standardized β	95% CI	t	p
Intercept	5.479	-	4.378–6.580	9.983	<0.001
NDI	0.190	0.668	0.126–0.255	5.929	<0.001
HADS-A	-0.134	-0.246	-0.257–0.011	-2.185	<0.001
Adjusted R^2^	0.377				

Disability: A stepwise multiple linear regression analysis was conducted to identify significant predictors of disability (NDI). The final model indicated a strong positive association with pain intensity (NRS), with β = 1.928 (t = 5.693, p < 0.001). This suggests that each one-point increase in pain intensity was associated with a 1.93-point increase in NDI score.

Additionally, the total HADS score (combined anxiety and depression) emerged as a significant predictor (β = 0.495, t = 4.246, p < 0.001), indicating that higher psychological distress was independently associated with greater perceived disability.

The model intercept was β = -6.993 (t = -2.540, p = 0.014), reflecting the estimated baseline disability level when both pain intensity and psychological distress are zero. This may imply an underlying perception of disability independent of measured physical or psychological factors.

The model explained 49.3% of the variance in NDI scores (adjusted R² = 0.493), indicating a strong predictive ability, although additional factors not included in the model may further contribute to perceived disability. All regression assumptions were satisfied for this model, including normal distribution of residuals, linearity, homoscedasticity, and absence of multicollinearity. Standardized beta coefficients are reported to facilitate the interpretation of predictor contribution (Table 6).

**Table 6:** Regression results for the variable NDI. NDI: neck disability index; NRS: Numeric Rating Scale; HADS total: Hospital Anxiety and Depression Scale – total score; Unstandardized β: unstandardized coefficient; Standardized β: standardized regression coefficient; SE: standard error; CI: confidence interval.

Variables	NDI				
	Unstandardized β (SE)	Standardized β	95% CI	t	p
Intercept	-6.993	-	-12.515–1.472	-2.540	0.014
NRS-pain intensity	1.928	0.549	1.249–2.607	5.693	<0.001
HADS total	0.495	0.410	0.261–0.729	4.246	<0.001
Adjusted R^2^	0.493				

## Discussion

This study aimed to identify predictive physical and psychological factors associated with pain intensity and disability levels in patients with neck pain at the point of initial physiotherapy evaluation. Identifying such factors is essential for guiding clinical decision-making and developing personalized rehabilitation strategies.

The findings demonstrated that increased pain intensity was associated with referred pain to the upper limbs, higher levels of disability (NDI >25/50), elevated depression scores, and, unexpectedly, with high levels of PA. Although the association between pain and depression was statistically significant, the effect size (r = 0.240) indicates a weak correlation. Clinically, this suggests a modest contribution of depressive symptoms to pain perception, which should be interpreted with caution when used to guide treatment decisions. Notably, pain was negatively associated with anxiety levels, which contradicts much of the existing literature and suggests the need for further investigation into the role of emotional processing and coping mechanisms in pain perception.

Disability levels were positively associated with pain intensity, anxiety, and depression, supporting the biopsychosocial model of pain and highlighting the multifactorial nature of perceived functional limitations.

However, due to the cross-sectional design of the study, causality cannot be inferred. The identified associations should be interpreted as correlational and hypothesis-generating rather than causal.

Previous systematic reviews on prognostic factors in neck pain emphasize the importance of using standardized tools to assess key variables such as range of motion (ROM) and BMI. Inconsistencies in variable selection and measurement across studies have likely contributed to inconclusive or conflicting findings regarding their predictive value [65]. Methodological standardization is critical for achieving consistent, comparable, and reliable results in clinical research.

Similarly, job satisfaction, although recorded through a validated questionnaire (MSQ), was not included in the final regression models, as it did not demonstrate significant associations with either pain intensity or disability in the univariate analysis. Given the sample characteristics and the acute-subacute symptom duration of a substantial proportion of participants, it is possible that occupational dissatisfaction had not yet exerted a measurable influence on perceived pain or functional limitations.

Finally, despite its theoretical relevance, CVA was not significantly associated with either pain or disability in our sample. This aligns with recent skepticism regarding its clinical utility as a standalone predictor in chronic neck pain populations.

Prediction of initial levels of pain and disability

Multivariable regression analysis was employed to determine the relative contribution of physical and psychological factors to initial pain intensity and disability levels. This approach allowed for the identification of the most influential predictors while controlling for potential confounding variables, thereby enhancing the interpretability and robustness of the results, even within a moderately sized sample [22,25]. 

Pain and Increased Disability

A moderate to strong positive correlation between pain intensity and disability was identified in the univariate analysis (r = 0.594, p <0.001). This finding supports the hypothesis that higher levels of perceived disability are associated with increased pain, consistent with previous studies indicating that functional limitations are often accompanied by heightened pain experiences [66].

In a study involving 90 patients with neck disability, pain intensity was identified as the strongest predictor of disability [67]. Similar associations have been documented in other musculoskeletal conditions, such as low back pain [68]. 

This link between pain and disability may be explained by protective behaviors and movement avoidance strategies [69], as well as psychological deterioration in patients with chronic neck pain [70].

Pain With Referral to the Upper Limb

The presence of referred pain to the upper limb emerged as an important factor associated with increased pain intensity. Patients reporting radiating symptoms experienced significantly higher pain levels compared to those with localized neck pain only.

This is consistent with previous findings comparing clinical outcomes in neck pain patients with and without upper limb referral, where pain intensity was numerically higher in the referred pain group, despite not reaching statistical significance (mean neck pain NRS: 5.69 vs. 7.22, p = 0.47) [71]. 

Pain and Activity Level

Statistically significant differences in pain intensity were observed among patient groups categorized by PA level. Patients with high PA levels reported the highest pain intensity scores, whereas those in the moderate PA group reported the lowest. 

Although this finding may initially appear counterintuitive, given the commonly accepted benefits of PA in musculoskeletal pain, excessive or unstructured PA may lead to physical overload and exacerbate symptoms [72]. This suggests that while PA is beneficial in general, it must be carefully tailored to individual tolerance levels in order to avoid overexertion and worsening of symptoms [73].

However, the direction and strength of this association were modest. These findings require further exploration in prospective designs.

Pain and Disability With Increased Depression Levels

Depression was found to be significantly associated with both pain intensity and disability. The correlation with pain was low to moderate (r = 0.240, p = 0.043), while the association with disability was moderate (r = 0.435, p < 0.001).

These findings are in agreement with previous literature suggesting a bidirectional relationship between depressive symptoms, neck pain, and disability [9,10]. Depression may influence pain perception through central mechanisms, including altered neurotransmission and increased pain sensitivity [74,75]. Furthermore, it is considered a mediating factor that can intensify the interaction between pain and functional impairment [76]. Consequently, the effective management of depressive symptoms in patients with chronic pain may contribute to reduced disability levels, underlining the multifactorial nature of rehabilitation for this population [8].

However, while depression contributed significantly to the prediction of disability (NDI), the standardized beta coefficients (β = 0.410) suggest a moderate effect. These predictors, although relevant, should be interpreted as part of a multifactorial framework rather than dominant, standalone contributors.

Disability and High Anxiety Levels

In line with the broader pattern of psychological influence, increased disability was also associated with higher anxiety levels (r = 0.387, p < 0.001). Anxiety has been linked to the amplification of pathological afferent pain input and elevated adrenaline levels, which may intensify the perception of neck pain and contribute to functional impairment.

These physiological and perceptual changes establish anxiety as a significant contributor to disability in patients with neck pain, reinforcing its role in the biopsychosocial model of chronic musculoskeletal conditions [70,77].

Pain Intensity Variance and Unexpected Anxiety Relationship

Multivariable regression revealed an inverse association between pain intensity and anxiety levels, whereby higher anxiety was linked to slightly lower reported pain. This counterintuitive finding contradicts much of the existing literature, which typically identifies anxiety as a pain-amplifying factor through central sensitization and emotional dysregulation mechanisms [8,78].

One possible explanation is the activation of coping strategies, such as avoidance, distraction, or cognitive reframing, that may accompany elevated anxiety in chronic pain patients. These mechanisms could modulate pain perception, resulting in lower reported intensity despite higher psychological distress.

According to the Gate Control Theory [79], psychological states can modulate nociceptive input at the spinal cord level. Anxiety may divert attention away from pain or increase endogenous inhibition, especially in individuals accustomed to living with persistent symptoms.

Furthermore, evidence suggests that chronic neck pain patients may not always report high anxiety, possibly due to long-term psychological adaptation, emotional detachment, or traits such as alexithymia [80]. In this study, most participants had chronic symptoms, which may have promoted the development of such coping patterns over time [81,82].

While this inverse relationship should be interpreted with caution, it underscores the complexity of psychological contributions to pain and highlights the need for individualized assessment beyond conventional assumptions.

Disability Variance

The multivariable analysis confirmed that disability (NDI) was positively associated with both pain intensity and psychological distress, reinforcing the bidirectional relationship between physical and psychological factors in individuals with neck pain [8,22,76,83]. While the regression model demonstrated a strong explanatory capacity (adjusted R² = 0.493), the negative intercept should be interpreted as a statistical artifact, a hypothetical value outside the range of observed data with no clinical implication.

These results highlight the strong interplay between pain and perceived disability. Anxiety and depression were significant predictors of higher disability scores and, when combined with pain, explained nearly 50% of the variance in disability outcomes.

Previous research has consistently shown that psychological distress exacerbates disability in neck pain patients [83]. Anxiety and depression increase the perceived difficulty in performing activities of daily living (ADLs), intensifying the functional limitations reported by patients [84]. The mechanism of catastrophizing, whereby patients overestimate the impact of pain and disability on their daily functioning, is closely linked to anxiety [85]. Depression is also strongly associated with chronic neck-related disability, particularly in individuals with long-standing functional restrictions in the cervical region [70].

Future perspectives

Future research should include studies with larger and more demographically diverse samples to enhance the generalizability of findings. The adoption of multicenter study designs and random sampling strategies is strongly recommended to reduce selection bias and better reflect real-world clinical populations. Subgroup analyses based on key patient characteristics, such as symptom duration, presence of referred pain, psychological distress profiles, or PA level, may provide more granular insight into individual response patterns and prognostic subtypes.

Longitudinal studies are essential to track changes in physical and psychological variables over time and to elucidate their temporal influence on rehabilitation outcomes, helping researchers distinguish causality from mere association. In addition, future studies should seek to validate these preliminary findings in independent cohorts and employ mechanistic approaches, such as neuroimaging or pain modulation assessment, to better understand how anxiety and coping strategies influence pain perception. The consistent use of validated patient-reported outcome measures (PROMs) across studies is also important to ensure cross-study comparability.

Moreover, future research should investigate potential moderators and mediators, including resilience, self-efficacy, and kinesiophobia, in the relationship between pain, function, and psychological burden. Finally, assessing the cost-effectiveness and feasibility of integrating psychological screening or tailored interventions into routine physiotherapy may inform best practices for managing neck pain. Overall, the complex biopsychosocial interactions identified in this study underscore the need for interdisciplinary and person-centered models in the management of chronic neck pain.

Advantages of the study

This study has several notable strengths that enhance its scientific and clinical relevance. First, it was conducted in a real-world physiotherapy setting, improving ecological validity and ensuring that the findings are applicable to everyday clinical practice.

Second, it simultaneously assessed both physical and psychological domains, reflecting the multidimensional nature of neck pain and supporting a biopsychosocial approach to rehabilitation. The inclusion of validated and widely used outcome measures, such as the NDI, HADS, MSQ, and IPAQ, adds further credibility and facilitates comparison with existing literature.

Third, all data were collected prospectively and prior to the initiation of treatment, providing a clear, unbiased snapshot of patients’ initial clinical profile. This timing minimizes the influence of therapeutic effects or recall bias.

Fourth, the use of multivariable regression modeling, combined with adequate sample-to-variable ratios, strengthened the statistical robustness of the findings and allowed for the identification of independent predictors of pain and disability.

Additionally, the emergence of an unexpected inverse relationship between anxiety and pain highlights the study’s capacity to reveal new hypotheses and underexplored dimensions of pain perception. Such findings underscore the value of including psychological variables even when their role may appear contradictory to conventional expectations.

Finally, the study focused on a primary care physiotherapy population, a setting often underrepresented in the literature, yet highly relevant for early-stage decision-making in musculoskeletal pain management.

Biases and limitations

This study presents several limitations that should be considered when interpreting the results. First, the sample was recruited via convenience sampling from a single physiotherapy clinic in Attica; thus, the sample consisted entirely of urban-dwelling Greek speakers, which limits the external validity and generalizability of the findings. The absence of random sampling increases the likelihood of response bias [86], while social desirability may have influenced patients’ responses during questionnaire completion [87]. Some participants had prior physiotherapy experience at the same facility, potentially introducing systematic bias through pre-existing beliefs or expectations regarding treatment outcomes [88]. Furthermore, the sample consisted exclusively of urban residents, limiting the applicability of findings to rural or more diverse populations.

Although the overall sample size was moderate, the use of multivariable regression adhered to accepted methodological guidelines for subject-to-variable ratios, minimizing the risk of overfitting [59]. However, the use of stepwise variable selection may still pose a risk of model instability and the exclusion of clinically relevant variables that did not meet statistical thresholds within this sample. Another limitation concerns the cross-sectional design of the study, with data collected at a single time point. This approach precludes causal inference and does not allow for tracking changes in physical or psychological variables over time or in response to treatment. Although the sample size followed commonly accepted guidelines for multivariable regression, no formal a priori power analysis was performed, limiting the ability to draw firm conclusions regarding the adequacy of the sample to detect small effect sizes.

Moreover, stepwise regression may inflate Type I error rates and is sensitive to sample-specific variance, potentially reducing the generalizability of the selected predictors. These limitations are particularly relevant in small to moderate sample sizes, where model overfitting and unstable variable inclusion may occur even when methodological ratios are met.

Furthermore, no correction for multiple comparisons (e.g., Bonferroni adjustment) was applied, given the exploratory nature of the analysis and the moderate sample size. This increases the risk of Type I errors, and the results should be interpreted with this limitation in mind. Also, the small size of some subgroups (e.g., severe anxiety n = 2; total disability n = 1) limits statistical power and may result in unstable estimates. Findings in these groups should be interpreted as hypothesis-generating rather than conclusive.

Another limitation concerns the absence of formal intra-rater reliability testing for the CVA in this study. Although all measurements were performed by experienced examiners following standardized procedures, and the Sit Screen module protocol of the PostureScreen Mobile® application was strictly applied, intra-rater agreement was not quantified. Previous studies have reported substantial to excellent intra-rater reliability for angular measurements related to CVA, such as head tilt and shoulder tilt, using the same tool in healthy populations. However, these findings cannot be directly generalized to seated individuals with neck pain, and this methodological gap should be addressed in future research.

Furthermore, the study relied entirely on self-reported measures, which may be subject to recall bias or misperception by participants. Important variables such as pain duration in weeks, medication use, workload intensity, and comorbidities were not assessed, despite their potential relevance in shaping pain perception and functional disability. Also, data collection procedures did not include blinding, which may have introduced observer or expectation bias. Finally, as this was a cross-sectional study, temporal relationships between predictors and outcomes could not be established, and therefore, no causal inferences can be made.

Additionally, other potentially relevant covariates were not measured (e.g., sleep quality, coping strategies, history of psychological interventions, work-related stress exposure). Their exclusion may have limited the ability to comprehensively capture the biopsychosocial profile influencing pain and disability in this population. Moreover, the study design did not allow for comprehensive control of all potential confounders. Although several relevant physical and psychological variables were included, others, such as sleep, coping style, occupational stress exposure, and comorbid conditions, were not fully accounted for. This limits the ability to isolate independent associations and should be addressed in future research through more robust adjustment strategies.

Despite these limitations, the study’s real-world clinical context, use of validated assessment tools, and integration of both physical and psychological domains offer valuable insights into the early-stage clinical profiling of individuals with neck pain.

## Conclusions

This study highlights the multifactorial nature of neck pain and related disability among patients entering physiotherapy. Pain intensity and disability were closely interrelated, and psychological variables, particularly anxiety and depression, emerged as significant contributors to perceived functional limitation. While depression showed consistent associations with both pain and disability, the strength of correlations was modest, and the role of anxiety proved more complex. Notably, the inverse association between anxiety and pain intensity contrasts with existing literature and may reflect adaptive coping strategies or emotional desensitization in chronic pain states. However, this finding should be interpreted with caution, as the cross-sectional design precludes any inference of causality. The direction and meaning of this association remain uncertain and warrant further longitudinal investigation. Additionally, other statistically significant associations, such as those involving PA or BMI, demonstrated only weak effect sizes, underscoring the importance of interpreting statistical significance within context.

These findings should be interpreted cautiously, considering methodological limitations including the cross-sectional design, limited sample size, and lack of adjustment for multiple comparisons. Future research is needed to clarify the mechanisms through which psychological variables interact with pain perception in real-world clinical settings. Emphasis should be placed on longitudinal data, multidimensional screening tools, and individualized rehabilitation approaches to optimize patient outcomes.
